# Self-organization and stability of magnetosome chains—A simulation study

**DOI:** 10.1371/journal.pone.0190265

**Published:** 2018-01-09

**Authors:** Bahareh Kiani, Damien Faivre, Stefan Klumpp

**Affiliations:** 1 Department Theory & Bio-Systems, Max Planck Institute of Colloids and Interfaces, Science Park Golm 14424 Potsdam, Germany; 2 Department Biomaterials, Max Planck Institute of Colloids and Interfaces, Science Park Golm, 14424 Potsdam, Germany; 3 Institute for Nonlinear Dynamics, Georg August University of Goettingen, Friedrich-Hund-Platz 1, 37077 Goettingen, Germany; Universitat Pompeu Fabra, SPAIN

## Abstract

Magnetotactic bacteria orient in magnetic fields with the help of their magnetosome chain, a linear structure of membrane enclosed magnetic nanoparticles (magnetosomes) anchored to a cytoskeletal filament. Here, we use simulations to study the assembly and the stability of magnetosome chains. We introduce a computational model describing the attachment of the magnetosomes to the filament and their magnetic interactions. We show that the filamentous backbone is crucial for the robust assembly of the magnetic particles into a linear chain, which in turn is key for the functionality of the chain in cellular orientation and magnetically directed swimming. In addition, we simulate the response to an external magnetic field that is rotated away from the axis of the filament, an experimental method used to probe the mechanical stability of the chain. The competition between alignment along the filament and alignment with the external fields leads to the rupture of a chain if the applied field exceeeds a threshold value. These observations are in agreement with previous experiments at the population level. Beyond that, our simulations provide a detailed picture of chain rupture at the single cell level, which is found to happen through two abrupt events, which both depend on the field strength and orientation. The re-formation of the chain structure after such rupture is found to be strongly sped up in the presence of a magnetic field parallel to the filament, an observation that may also be of interest for the design of self-healing materials. Our simulations underline the dynamic nature of the magnetosome chain. More generally, they show the rich complexity of self-assembly in systems with competing driving forces for alignment.

## Introduction

The cytoskeleton provides a mechanical scaffold for the cell and it is a key player in organising the cellular components [1], While originally considered to be unique to eukaryotes, the important roles of the cytoskeleton are now also recognized for bacteria [2–5]. In addition to contributing to the global cellular structure and to the reorganization of that structure during cell division, cytoskeletal filaments also have roles in highly specific functions. One such example is the magnetosome filament in magnetotactic bacteria, which scaffolds the magnetosome chain that provides these cells with a magnetic moment. The magnetosome chain acts as a compass needle and allows these cells to navigate in the magnetic field of the Earth [6]. Magnetosomes are membrane-enclosed organelles that contain magnetic nanoparticles. The magnetosomes are attached to a cytoskeletal filament based on the actin-related protein MamK to form a chain-like assembly that provides the cell with the maximal magnetic moment [7]. In addition to providing a scaffold for the assembly of the magnetosomes, the MamK filament is also involved in the reorganization of the magnetosome chain during cell division [8]. As a scaffold, the filament stabilizes the magnetosome chain against ring closure, which is favored by the magnetic interactions of the magnetosomes, but would be detrimental for the function of the chain [9]. Thus, the organization and the mechanical stability of the magnetosome assembly are directly linked to its function as a cellular navigation device. Since the structure of that assembly involves magnetic interactions, it can be perturbed in a non-invasive fashion using external magnetic fields. Indeed, magnetic fields have been applied to magnetotactic bacteria immobilized in a gel to probe the mechanical properties of the magnetosome filament. Broken magnetosome chains have been observed after the application of a sufficiently strong magnetic field that was rotated with respect to the chain axis, such that the interaction of the magnetic moments of the individual magnetosomes with the external field competes with their interaction with each other [10].

In this study, we propose and study a model describing the structure of magnetosome chains. Our model describes the magnetic interactions of the magnetosome particles, both with each other and with an external magnetic field, as well as binding of the magnetsomes to the filament. It thus provides a framework for studying the self-assembly and the stability of magnetic particles in the presence of a filamentous structure that stabilizes chain structures. Self-assembly of magnetic nanoparticles is currently studied quite extensively and a large number of different possible structures have been found [11–14]. We therefore start with simulations of the self-assembly of magnetosome particles in the absence of a filament and characterize the different equilibrium structures that we observe, and study the effect of an external magnetic field and of binding to the filament. Then, we use our model to probe the mechanical stability of the magnetosome chain with a magnetic field that is rotated with respect to the chain axis/filament axis, as in the experiment by Koernig et al. [10]. We characterize the rupture of the magnetosome chain, which we find to occur via two subsequent transitions. Finally, we simulate the recovery of broken magnetosome chains. The results of our simulations underline both the mechanical stability and the dynamic nature of magnetosome chains.

## Materials and methods

### 0.1 Model

In our modelling approach, the magnetosome chain is described as a linear chain of *N* spherical magnetic particles that are connected to a rigid filament by elastic connections. The magnetic nanoparticles are taken to consist of magnetite with the saturation magnetisation (per volume) of 0.48 × 10^6^ Jm^−3^ T^−1^ at room temperature [15]. For a particle of radius *R* = 20 nm, a typical value for magnetosomes in the well studied *Magnetospirilla* species [16], the magnetic moment is thus *m* = 1.61 × 10^−17^ JT^−1^. Magnetosome particles in this size domain are in the single-domain regime, i.e. they have permanent magnetic dipoles with a rather large magnetisation due to the absence of magnetic domains [17]. The minimal distance between neighbouring magnetosomes is *d*_*n*_ = 2*R* + *d* ≃ 50 nm, where *d* ≃ 10 nm is a gap distance between the magnetic particles accounting for the surrounding membranes. Two particles *i* and *j* interact through the potential *E* = *E*_dd_ + *E*_hc_. Here *E*_hc_ is the hard-sphere potential given by

$$\begin{array}{c} E_{\text{hc}}=\{\begin{array}{cc} 0 & \text{if}\;r_{ij}⩾d_{n}\\ \infty & \text{if}\;r_{ij}<d_{n}. \end{array} \end{array},$$ (1)

where *r*_*ij*_ is the inter-particle distance. *E*_dd_ is the dipole-dipole interaction,

$$\begin{array}{c} E_{\text{dd}}=-\frac{\mu_{0}}{4\pi }\frac{1}{r_{ij}^{3}}(\frac{3\left(m_{i}\cdot r_{ij}\right)\left(m_{j}\cdot r_{ij}\right)}{r_{ij}^{2}}-m_{i}\cdot m_{j}), \end{array}$$ (2)

where *μ*_0_ = 4*π* × 10^−7^NA^−2^ is the vacuum permeability, the **m**_*i*_ and **m**_*j*_ are the magnetic moments of the dipoles and the **r**_*ij*_ are the distance vectors between them, with *r*_*ij*_ = |**r**_*ij*_|. In the following, we will assume that all dipoles have equal absolute value, |**m**_*i*_| = *m*. They are linked to the filament through cable-like, semi-linear springs with elastic energy

$$\begin{array}{c} E_{\text{elasticity}}=\{\begin{array}{cc} +\frac{1}{2}k_{l}{\left(l-l_{0}\right)}^{2} & \text{if}\;l>l_{0},\\ 0 & \text{if}\;l\leq l_{0}. \end{array} \end{array}$$ (3)

Throughout this work, the rest length is taken to be *l*_0_ = 5 nm, a typical molecular length and the spring constant is set to *k*_*l*_ = 0.106, a value similar to that of another linker to cytoskeletal filaments, the molecular motor kinesin [18]. *l* is the distance between the attachment points of the linker on the surface of the particle and on the filament.

In addition, the linkers have a discrete degree of freedom indicating their bound or unbound state. At any position in the cell, particles can thus be in two states, bound to the filament or unbound. We should note that, there is a specific binding between magnetosome membrane and the filament mediated by the MamJ protein [7, 19]. Since several of these MamJ proteins are present, we assume the binding can form at any point on the membrane of the magnetosome particle and the filament. Switching to the bound state, a particle gains a negative binding energy *E*_b_ (with a default value of −2*k*_B_*T*) plus the (typically positive) elastic energy of the cable at length *l*. In addition, the magnetosome particles may interact with an external magnetic field, **B**, with interaction energy

$$\begin{array}{ccc} E_{B}= & & -\sum_{i=1}^{i=N}m_{i}\cdot B\\ = & & -\sum_{i=1}^{i=N}mB\cos\left(\theta_{B}-\theta_{i}\right), \end{array}$$ (4)

where the second expression is valid for a field under an angle *θ*_*B*_ with the axis of the magnetosome chain.

### 0.2 Computer simulation

#### 0.2.1 General approach

We carry out Monte Carlo simulation for *N* = 20 magnetosome particles in a cylindrical simulation box, in which the filament extends along the main axis of the cylinder. At every time step of the simulation, we perform a Monte Carlo move for a randomly chosen magnetosome particle. Three types of moves are possible: a spatial move, a change in orientation of its magnetic moment or a change in its attachment state (bound to unbound or vice versa). The moves are accepted according to a Metropolis criterion [20].

The spatial movements of magnetosome particles are performed by changing the position of magnetosome particles with a random vector whose *x*, *y*, *z* components attain random values between −*d*_*n*_ to *d*_*n*_. To simulate the magnetic moment, we attribute to each magnetosome particle a vector with the fixed absolute value *m* and an orientation which undergoes random polar and azimuthal changes between −5 and 5 degrees. We assume that the dipole moment of a magnetosome has a fixed orientation with respect to the inner coordinate system of the particle, thus any change in the orientation of the dipole causes a rotation of the corresponding particle.

#### 0.2.2 Simulation of equilibrium structures

One of the difficulties in the simulation of self-assembly of magnetic particles is the fact that the structures that are formed are located in the local minima of the energy landscape and once these structures are formed, the breaking of their magnetic bonds becomes very unlikely, which prevents the finding of the global minimum structure. This problem is most cumbersome when we are looking for structures at low temperature for which the thermal fluctuations are several orders of magnitude weaker than the magnetic interactions (in our case, typically 1000-fold) so that at this temperature a structure in the local minimum could become frozen. To overcome this difficulty, we apply simulated annealing, a simulation method in which, starting from a large value, the temperature is slowly reduced during the course of a simulation in order to reach the global minimum [21]. Simulated annealing is not guaranteed to converge to the exact minimum of the energy and if we want to extend the algorithm to finite temperature different runs will give different results [22], but it can be used to overcome the problem of frozen structures. At the same time, by moving the system into and out of different regions of the phase space, more diverse configurations are sampled, increasing the probability of finding the true minimum.

## 1 Results and discussion

### 1.1 Equilibrium configurations of magnetic particles

We first consider magnetic particles in the absence of an external magnetic field. We start with the randomly distributed magnetosome particles and follow the formation of minimal-energy structures using simulated annealing and without binding to the filament. This scenario resembles the situation in cells of the mutant Δ*mamJ*, as we will discuss below.

**Fig 1:**
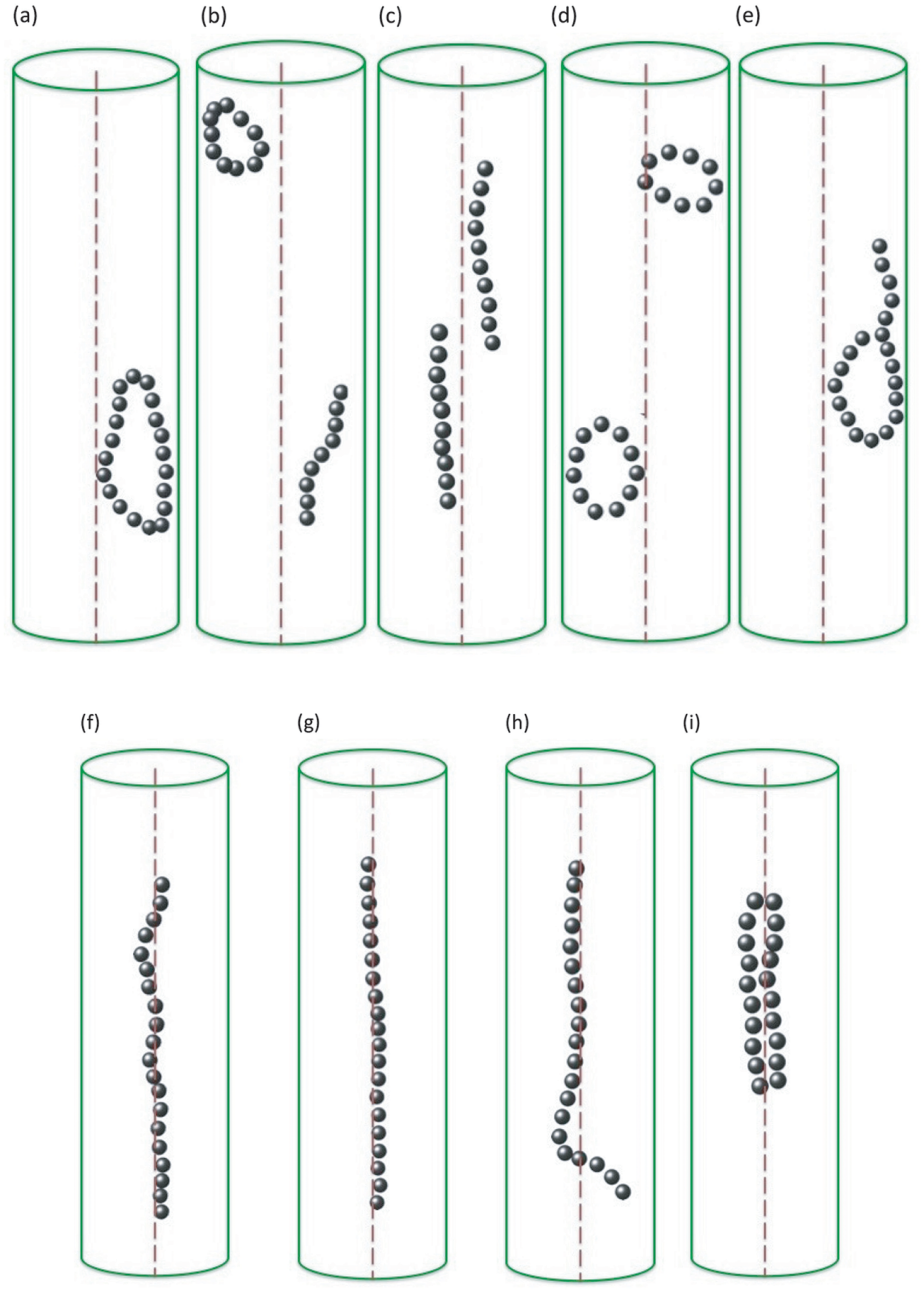
Local minimum structures observed in the Monte Carlo computer simulation of 20 randomly distributed magnetosome particles with no binding energy to the filament (a–e). Observed structures include chains (b, c), rings (a, b and d) and handle (e). In the presence of the binding energy to the filament, particles form semi-linear structures along the filament (f–i).

The local minima found in this way exhibit a variety of structures, such as linear chains, closed rings and handle like structures, as shown in Fig 1(a)–1(e). Among those, chains and rings are the most common structures found in our simulations. A transition between these two types of structures is characterised by an interplay between the energetic gain of closing the rings, the energetic cost of chain bending [9] and the higher entropy of the linear structures (a minor contribution because of the low temperature). However, the energy difference between the two structures is rather small. The other structures that we observe can be interpreted as combinations of linear chains and rings, such as two chains, two rings or a chain and a ring. Handle structures also fall in this class as they can be interpreted as a combination of a chain and a ring sharing one of the particles at the end of the linear chain. Chains, rings and handles have been observed in experiments with isolated magnetosomes, [23], as well as other magnetic nanoparticles [11, 13].

**Fig 2:**
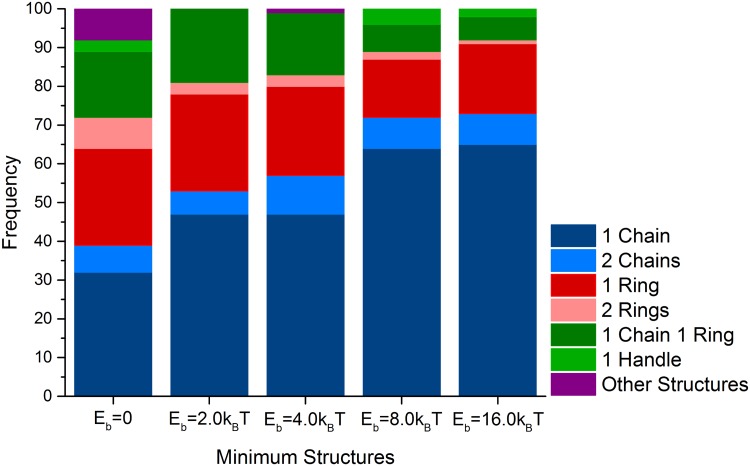
Fraction of configurations observed in the Monte Carlo computer simulations in the absence and presence of different values of the binding potential to the filament. In each case configurations were extracted from 100 runs of the simulations. Simulations classified as Other structures include open rings (observed in the case of *E_b_* = 0*k*_B_*T*) and a single ring accompanied by two chains (in the case of *E_b_* = 4.0*k*_B_*T*).

To quantify the frequency of the different structures, we performed 100 runs of the Monte Carlo simulation with simulated annealing. The results are shown in Fig 2 and confirm the predominance of the formation of chain and ring structures with nearly the same frequencies while handle-like structures account only for 3% of the structures observed.

In their native environment, the cytoplasm of magnetotactic bacteria, magnetosomes are usually attached to a cytoskeletal filament, a case that we will discuss next. However, there are mutants lacking either the protein MamK, the main component of the filament, or the protein MamJ, which links the magnetosomes to the filament [7]. In the absence of MamK, straight magnetosome chains are still formed, but they are not properly positioned in the center of the cell, they are shorter than in the wild type and fragmented chains or multiple chains in one cell are observed [24]. Thus, the linear structures we see in our simulations are also observed in this mutant, however, ring closure is not observed. In the absence of MamJ, the linear structures disappear, and random clusters of magnetosomes are observed rather than rings [7, 25]. Such clusters are not seen in our simulations. However, the different phenotypes of MamK and MamJ deletions suggest that filament attachment may not be the whole function of MamJ and MamK. It is possible that additional interactions, upon direct contact between the membranes of two particles (or mediated by proteins in the membrane) contribute to cluster formation. In fact, clusters can be observed if additional attractive interactions between the particles are included or if the density of the magnetic particles is strongly increased. Both observations are consistent with earlier simulation results that showed cluster formation at higher densities [26] as well as a strong dependence of pattern formation on short-range interactions in addition to the magnetic dipole-dipole interactions [27].

Now we include the binding of particles to the filament in our simulations. In that case, we predominantly obtain one single linear chain of magnetosome particles formed along the filament with magnetic moments in the direction of the chain. Some representative examples are shown in Fig 1(f)–1(h). We quantify the frequency of the different structures in Fig 2 for different values of the particle-filament binding energy. Chains and rings are found to be the dominant structures formed. With increasing binding energy, more chains (up to about 2/3 of the cases) and fewer rings are observed. For the highest binding energies we simulated, *E_b_* = 8.0*k*_B_*T* and *E_b_* = 16.0*k*_B_*T*, all the ring and handle-like structures are assembled around the filament. In the case of *E_b_* = 16.0*k*_B_*T*, these structures are typically formed by two antiparallel chains that are bound to two sides of the filament and converge at their ends into a ring or handle-like structure, Fig 1(i).

Next, we consider the effect of an external magnetic field as used in recent experiments with immobilised cell [10]. If the external field has a non-zero angle relative to the direction of the filament, alignment of the dipoles with the field competes with the alignment with other dipoles bound to the filament. The binding energy favours configurations with all particles close to the filament, while the external magnetic field-dipole interaction favours alignment of the magnetic dipoles with the external field, rotating them and increasing their elastic energy. For strong fields and strong magnetic interactions, one may even expect chains of particles in the direction of the field and detached from the filament. Thus, dependent on the strength and angle of the external field, and on the binding energy and stiffness of the linkers, the magnetosomes will adopt new configurations. In fact, for sufficiently strong fields, we mostly observed configurations with multiple chains that are tilted away from the filament in the direction of the field but are attached to the filament via their middle particles.

**Fig 3:**
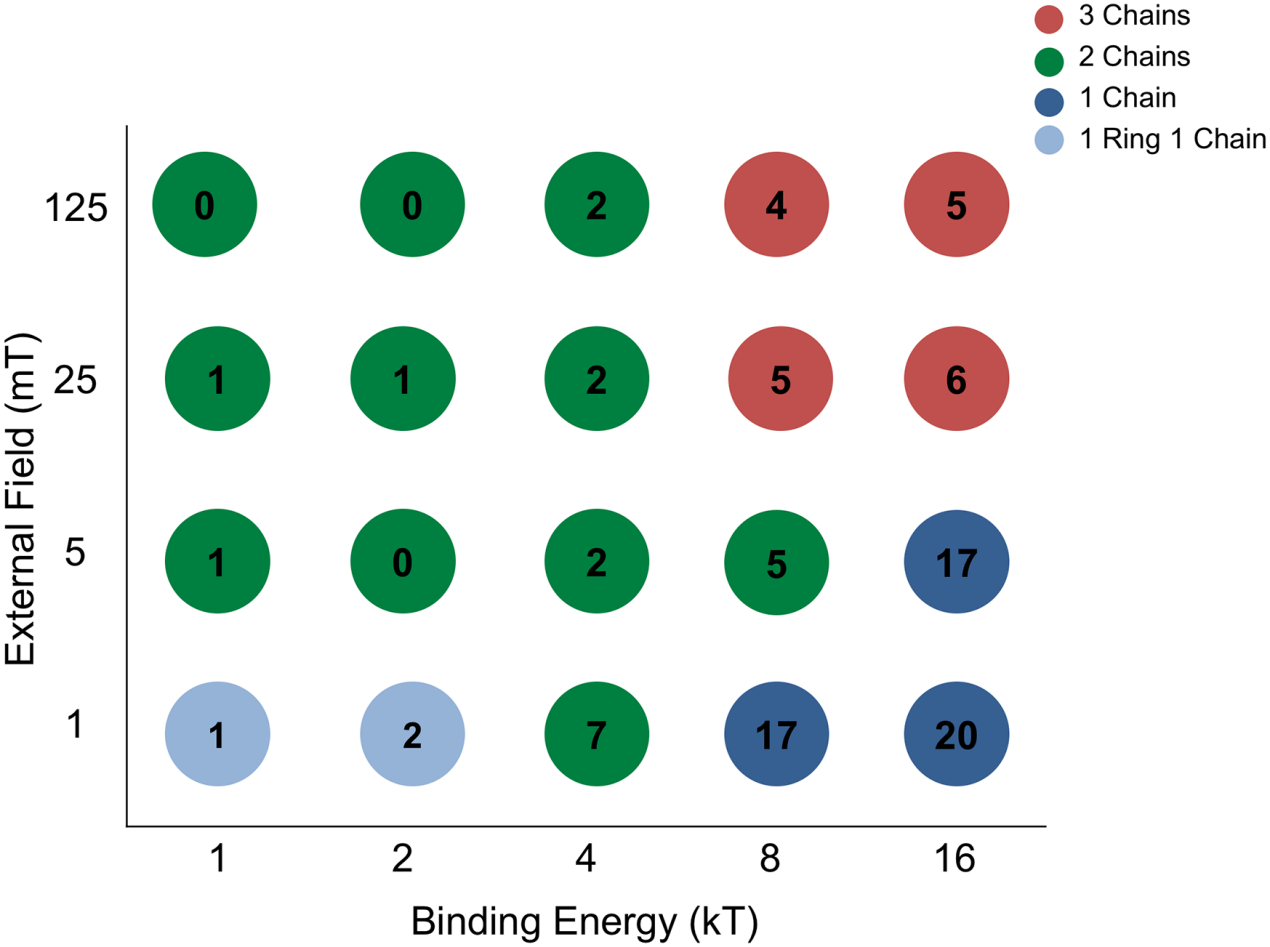
Final configuration of magnetosome particles for different values of the binding energy to the filament and the external magnetic field strength. The external field is fixed to an angle 90°, relative to the direction of the filament. The color coding indicates the magnetosome configuration quantified by the number of chains (average of 5 simulations, rounded to integers). The number in each circle indicates the number of particles bound to the filament (also averaged over 5 simulations and rounded to integers).

Focusing on the competition between the binding energy and the external field, we performed systematic Monte Carlo simulations for different values of the binding energy and the external magnetic field strength under a fixed angle of 90° relative to the direction of the filament. For each combination of the binding energy and the external magnetic field strength, we measured the number of chains formed and the number of magnetosome particles that are bound to the filament. The results of these simulations are presented in Fig 3. For the lowest external field measured, *B* = 1 mT, we typically observe a single chain. The average number of bound particles (that connect the chain to the filament) increases with increasing binding energy to the point that for *E*_*b*_ = 16*k*_*B*_*T* all the particles of the chain are bound to the filament. For the opposite limit of the high external field (*B* = 125 mT), we always observe multiple chains, oriented in the direction of the field and either not attached to the filament or attached via particle(s) in the middle of the chains, depending on the binding energy. The latter configuration reflects a competition between several energy contributions: The presence of multiple chains is favored by the additional binding energy of their middle particles to the filament and the alignment of the magnetic moments with the external field, but a single chain configuration is favoured by the additional nearest neighbour interactions (≃ 100*k*_*B*_*T*). In fact, parallel magnetic moments of the chains (in the multiple chain configuration) result in repulsive interactions between chains and build an energy barrier between two configurations which stabilises the multiple chain configuration kinetically. For weaker external fields, the nearest neighbor interactions become dominant over the alignment with the field, resulting in the formation of the single chain configuration. At the same time, it is less difficult to disorient the parallel magnetic moments resulting in a lower energy barrier to single chain formation.

### 1.2 Probing magnetosome chain mechanics by rotating an external field

**Fig 4:**
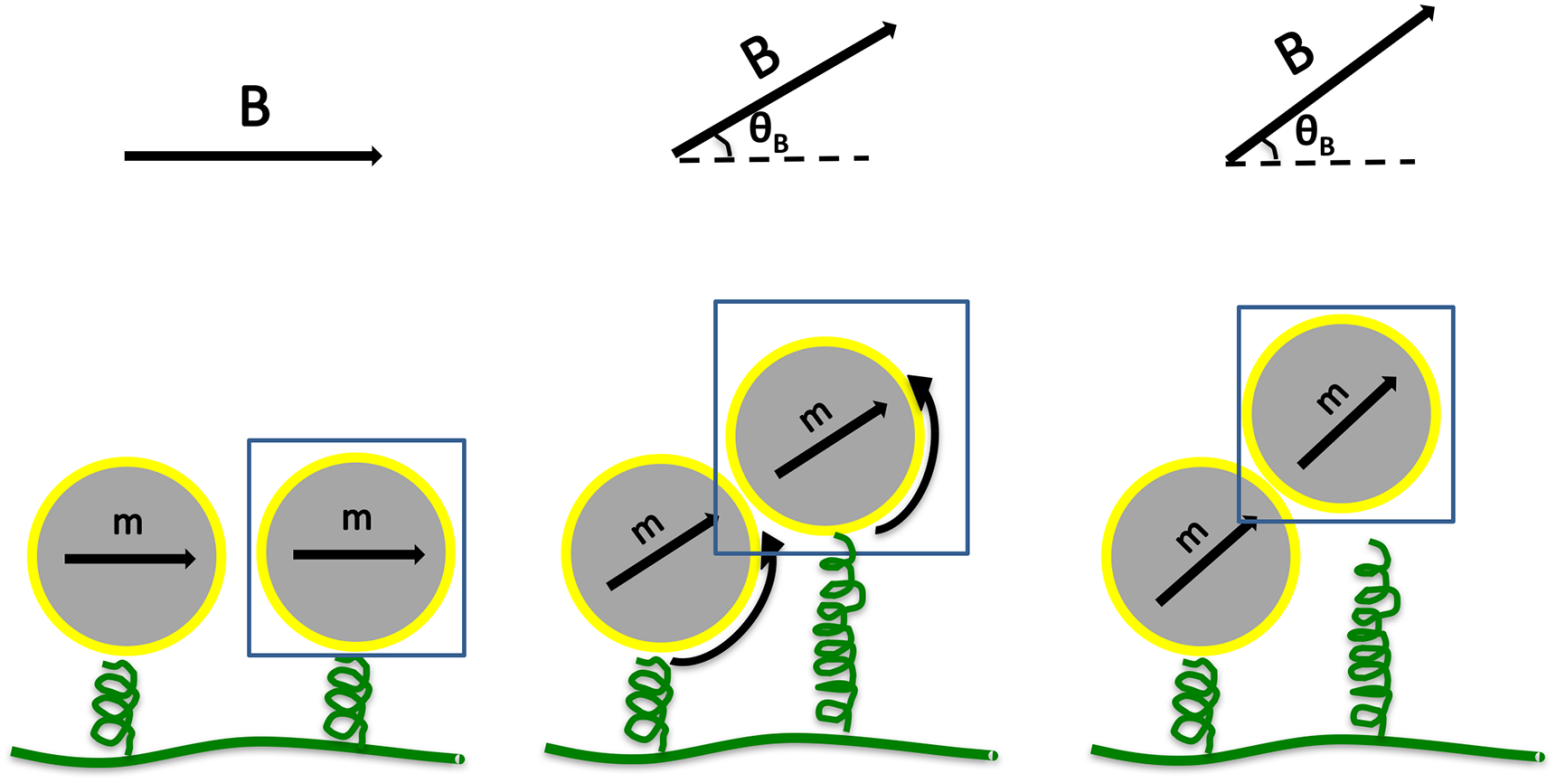
Schematic view of a magnetosome particle (shown in blue frame) connected to the filament in the presence of dipole-dipole interaction and an external magnetic field with the angle *θ*_*B*_ relative to the direction of the filament. At the threshold angle of *θ*_*B*_ ≃ 27°, the binding energy rises higher than the elastic energy and therefore the binding to the filament is not anymore an advantage.

The mechanical stability of the magnetosome chain was recently probed in living magnetotactic bacteria by Koernig et al. [10]. In this work, by rotating a magnetic field away from the magnetosome chain axis in immobilised bacteria, the magnetosome chain structure was set under tension and eventually, the linear structure ruptured. This experiment was done at the population level showing the average behaviour of a large number of cells. With our simulations, we can perform the corresponding *in silico* probe for individual magnetosome chains. Thus, we consider a magnetosome chain in an external magnetic field at an angle *θ*_*B*_ relative to the direction of the filament. This angle is rotated from 0° to 90° about an axis perpendicular to the plane of the magnetosome chain. By rotating the external field to a non-zero angle, we exert a mechanical force on the linkers of the particles to the filament. This is illustrated in Fig 4. Considering this simple scenario, we can estimate the critical angle at which, due to the pull on the magnetosomes, the linkers to the filament break. For two particles, we can see that if we rotate the external field enough, we generate a force on the linker of the particle to the filament to the limit that binding to the filament is not any more favourable. We calculate this explicitly in S1 Appendix.

**Fig 5:**
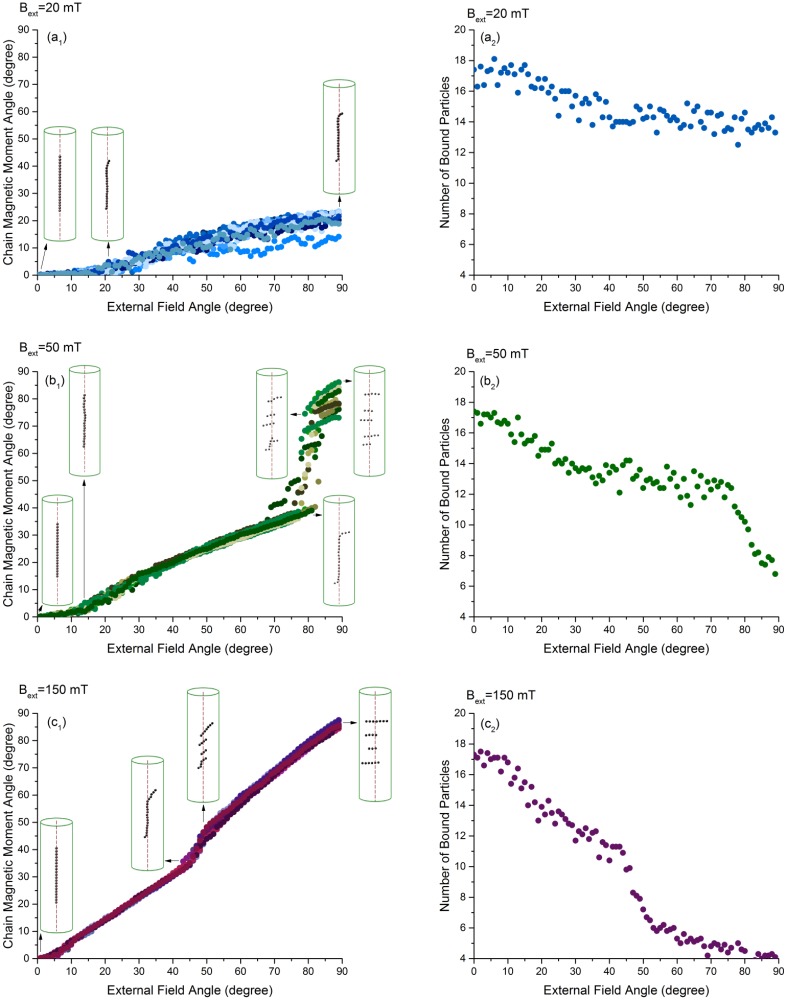
Response of the chain magnetisation to a rotation of the external field: a_1_, b_1_ and c_1_ show the change in the orientation of the chain magnetic moment in different angles and strengths of the external field. How the average number of magnetosome particles bound to the filament changes during the rupture is presented for each field strength in a_2_, b_2_ and c_2_. In each plot, different colours represent 10 trajectories extracted from Monte Carlo simulations.

For a more detailed study of the stability of the whole magnetosome chain, we performed Monte Carlo simulations for different field strengths (20 mT, 50 mT, 150 mT) and changed the orientation of the field (angle *θ*_B_) in a stepwise manner, increasing the angle by 1° every 10^+7^ Monte Carlo steps, from 0° to 90° relative to the direction of the filament. We measured the orientation of the magnetic moment (angle *θ*_m_) of the magnetosome chain and the number of bound particles as a function of the angle, Fig 5.

For weak field strength (20 mT), the magnetic moment of the chain follows the external field only weakly, reaching a maximal deflection of *θ*_m_ ≃ 20°. This change in the angle results mostly from the particles at both ends of the chain. These are pulled away from the filament by the rotation of the field, detach from the filament and orient in the direction of the field. Unbinding of the particles at the end occurs at the critical angle, *θ*_B_ ≃ 20°. Other than that, the linear arrangement of the particles on the filament is not disrupted.

For the field strengths 50 mT and 150 mT, rupture occurs through two discrete events at different critical values of the angle between the field and the filament. At the first critical angle (≃ 10° and ≃ 3° for the field strength of 50 mT and 150 mT, respectively), the two ends of the chain unbind from the cytoskeletal filament and tilt away from the chain axis toward the direction of the external field as seen for the case of 20 mT. At the second critical angle (≃ 77° and ≃ 45° for the field strengths of 50 mT and 150 mT, respectively) the chain fragments into smaller pieces that are oriented in the direction of the field and are bound to the filament through their two middle particles.

**Fig 6:**
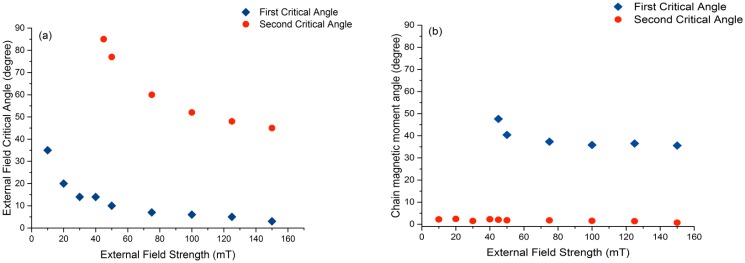
(a) The external field critical angles for chain rupture: As field strength increases, the magnetosome chain ruptures at lower values of the external field angle. (b) Chain magnetic moment angle at first and second critical angles of the external field as a function of the field strength. The figure shows the independence of this angle from the field strength.

When we use a stronger field, the magnetosome particles are rotated to larger angles increasing the force pulling their linkers in early steps. Therefore rupture occurs at smaller angles of the external field (smaller critical angles) and at the rupture point the transition in the angle of the chain magnetic moment is smaller. The shift of the two critical angles as a function of the field strength is shown in Fig 6. While the first (lower) critical angle is present for all field strengths we simulated, the second critical angle is only seen for fields of 45 mT and higher.

The rupture process is stochastic and shows some variation between different runs of the simulation, in particular for the intermediate field strength of 50 mT, where the second rupture step occurs in a window of approximately 7°. We tested whether this variability is due to dynamic competition between the rate at which the angle *θ*_*B*_ increases and the rate of the rupture process, but simulations in which the angle was varied two-fold faster or slower showed very similar results.

The key role in the chain rupture is played by the competition between the dipole-dipole interactions and the interactions of the dipoles with the external field. If the field direction is parallel to the chain, both these interactions will have the minimum energy. By rotating the external field, relative to the direction of the filament, the interaction with the external field drives the dipoles toward an alignment with the external field, while the dipole-dipole interactions favour a dipole orientation along the chain axis. Rotating the dipoles, thus increases the dipole-dipole energy, up to the point, where the attractive dipole-dipole force becomes repulsive. At this point, the magnetosome chain breaks into pieces. However, the two particles at the ends, due to the weaker dipole-dipole stabilisation, follow the external field more freely, giving rise to the first critical angle. Fig 6(b) shows that the angle of the magnetic moment of the chain seen at the critical angles of the external field is independent of the external field strength. This observation indicates that it arises from the dipole-dipole interactions. The change in the state of the linkers of the particles to the filament can be followed more closely by looking at the number of particles bound to the filament during the rupture process, as shown in the right column of Fig 5. At the critical angles, steep changes in the number of bound particles are observed.

**Fig 7:**
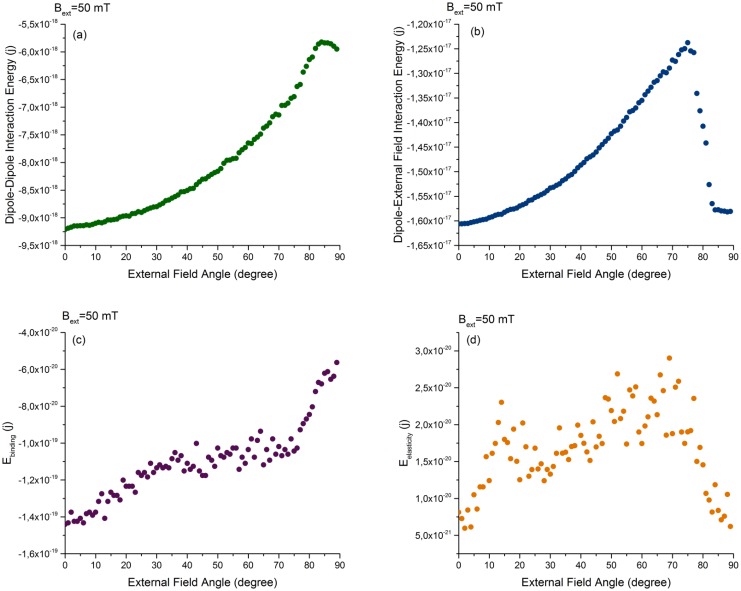
Behaviour of the dipole-dipole interactions energy between magnetosome particles (a), the external magnetic field-dipole interaction energy (b) interaction energies of magnetosome particles, the binding (c) and elastic energies (d) of the linkers of the particles to the filament in the magnetosome chain under an external magnetic field of 50 mT for different angles of the external magnetic field relative to the direction of the filament.

Fig 7 shows how the individual energy contributions change during the rupture process. After rupture, the dipoles in each smaller chain are aligned with each other, but from one chain to the other they are parallel. Therefore the dipole-dipole interaction decreases but not to the initial minimum, as shown in Fig 7(a). However, as the dipoles in the smaller pieces are all aligned with the external field, their interactions with external magnetic field decreases to its minimum value, see Fig 7(b). The non-zero values of binding and elastic energies are due to the attachments of particles in the middle of the chains to the filament (Fig 7(c) and 7(d)). The observation of a slowdown in the slope of the binding and elastic energy after the first critical angle is explained by the fact that the detached particles do not contribute any longer to the binding and elastic energies.

Comparing our results with the experiments of Koernig et al. [10], we observe overall qualitatively similar behavior, but also some striking differences. In the experiments, the particle orientation (observed through the orientation of crystal planes in the X-ray diffraction pattern), follows the direction of the external field almost exactly for field strengths exceeding a threshold. The threshold, 35 mT is comparable to the threshold value of 40 mT beyond which the second critical angle (characterizing chain rupture) is observed in our simulation. However, in our simulations, the particle orientation does not follow the field angle as closely as in the experiments. Correspondingly, the threshold field strength in our model is predicted to be angle dependent (interpreting the second critical angle in Fig 6 as a critical field strength). Likewise for low field strength, the experimental particle orientation seems not to follow the external field at all, while in our model some rotation is still seen. This is also the case in the much more coarse-grained model of Koernig et al. While we cannot exclude that additional dynamic processes not included in our model contribute to the experimental observations, we expect that the main reason for these differences is that experimental data are for bulk samples, while our current simulation is for a single cell. In bulk samples, parameters such as number and size of magnetosome particles or the distance between neighbouring particles are not fixed, but rather vary from cell to cell according to some distribution. Therefore, we expect the experimental observation to reflect an average over these distributions and hypothesize that some of the finer details of the single-cell trajectories may be averaged out during this process.

**Fig 8:**
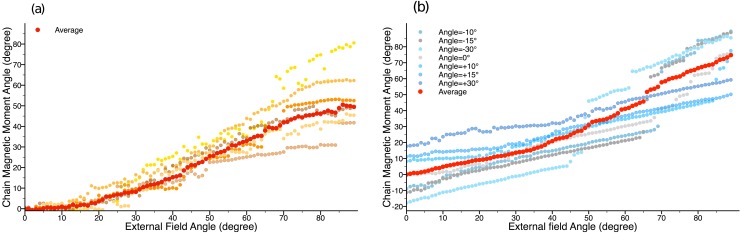
Bulk-sample-like simulations of magnetosome chain rupture. For chains (a) with particles with different sizes and (b) with different orientations relative to the initial orientation of the external field.

To test this hypothesis, we consider two scenarios based on experimentally observed variability. We first simulate chains with a distribution of particle sizes. The distribution is taken to be Gaussian with mean 20.3nm and standard deviation 6.8nm, an approximation to the distribution determined from electron microscopy images [28]. Individual chains are seen to rupture at different angles with more or less pronounced abrupt transitions. The average (taken here over 10 such runs) tends to smoothen out these abrupt transitions (Fig 8(a)). In addition, we simulate chains that are imperfectly aligned with the initial external field, due to imperfect alignment of the cells in the sample, as observed recently [29]. Fig 8(b) shows simulation results for chains with different orientations, the average of these, weighted with a Fisher distribution [29], again smoothens out the abrupt transitions.

### 1.3 Recovery of the chain after disruption

**Fig 9:**
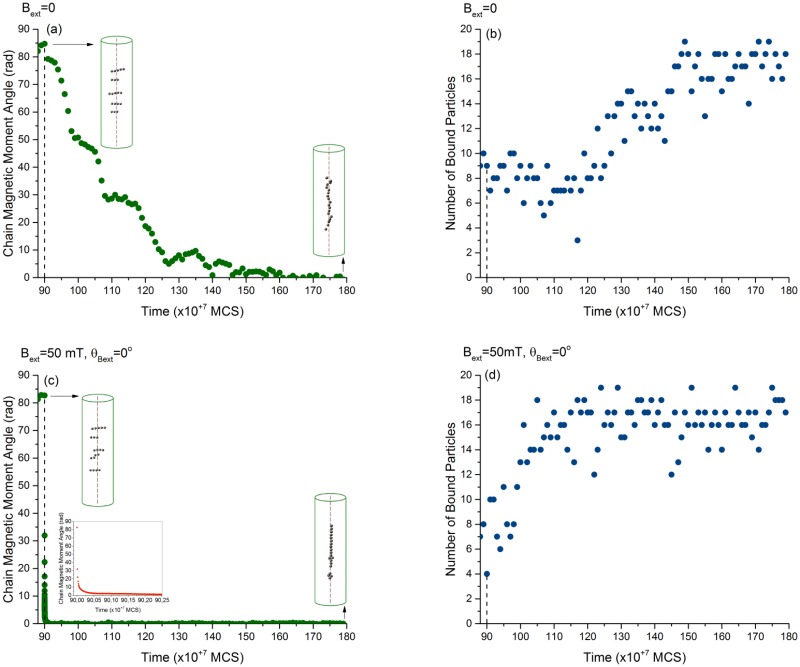
Behaviour of the magnetosome chain after the disruption with an external magnetic field, from 90 × 10^+7^ MCS to 180 × 10^+7^ MCS. (a, c) Magnetic moment of the chain and (b, d) number of particles bound to the filament after elimination of the external field (a, b) and after treatment with an external field aligned with the direction of the filament (c, d). The inset on (c) displays the zoomed-in behaviour of the chain directly after the alignment of the external field with the filament. MCS refers to Monte Carlo Steps.

Finally, we consider the chain dynamics after rupture. As above, we perform simulations in which the angle of the external field with respect to the filament is increased from 0° to 90°. After the chain rupture, we observe the recovery of the chain in two scenarios: In one case, the magnetic field is turned off after the angle of 90°, in the other, the field angle is set back to zero such that it is aligned with the direction of the filament. These simulations show that in both scenarios, a linear chain reassembles along the filament (Fig 9). That chains do recover from the breakage due to a magnetic field under a non-zero angle with the chain axis or filament axis, is also indicated by the experiments of Koernig et al., where broken chains were observed for cells that were crosslinked after exposure to a sufficiently strong field, but wild-type chain structure for cells that were not cross-linked [10]. Our simulations results thus provide additional support for the rather dynamic nature of the magnetosome chain suggested by these experiments.

Even though recovery is seen in both simulated scenarios, its dynamics is quite different for the two cases: If reassembly of the chain occurs with the field switched off, the recovery of the magnetic moment of the chain and of the number of particles bound to the filament takes about 50 × 10^7^ Monte Carlo steps. In the second case, i.e. in the presence of a magnetic field parallel to the filament, recovery is faster. Moreover, the magnetic moment and the number of bound particles recover on very different time scales. For the magnetic moment, the recovery is about 50 times faster than with the field switched off, while the recovery of the number of bound particles is only about 3 times faster. In fact, one barrier that fragmented chains should overcome to reassemble on the filament is the repulsive dipole-dipole interactions. The interaction between the dipoles and the magnetic field aligned with the direction of the filament facilitates this transition. To a lesser extent this effect can also be seen in the number of bound particles, while in the absence of a field displays a lag period before the recovery, likely because the particles have the reach the filament by diffusion. This lag is is absent in the presence of a magnetic field, where the number of bound particles increase immediately after the field is reset in the direction of the filament.

## Conclusion

We have developed a theoretical framework to study the structure of magnetosome chains in magnetotactic bacteria, which enables us to address their energetics, stability and mechanical properties. Our model describes the magnetostatic interactions between magnetosome particles and between magnetosome particles and an external magnetic field and the binding of the magnetosome particles to the cytoskeletal filament via elastic linkers.

With this model, we investigated the assembly of initially randomly distributed magnetosome particles and the resulting equilibrium configurations. Our results presented a variety of different equilibrium configurations including linear chains, closed-rings and handle-like structures. The relative stability of chain and ring structure, which are the most frequent ones, reflects the energy cost due to the bending of the chain, as well as an entropy loss and the gain of an additional dipole-dipole interaction upon chain closure [9]. Binding to the filament stabilizes the linear chain structure with all magnetosome particles bound to the filament. In our simulations, robust formation of a magnetosome chain requires sufficiently strong binding to the filament. We then studied the impact of an external magnetic field. Depending on its strength and direction as well as on the magnetosome binding to the filament, single or multiple chains attached to the filament in the middle of the chain were observed. Configurations with multiple chains are kinetically stabilized by repulsive dipole-dipole interactions between short chains that are oriented parallel to each other. The transition from a linear chain of magnetosomes along the filament to this ruptured configuration with several short chains, which reflects the competition between alignment of magnetic moments with each other or with the external field, can be used as a probe for the mechanical stability of the magnetosome chain and has been used experimentally to that end by Koernig et al. [10] Here we have performed the corresponding experiment in silico and shown that rupture occurs through two consecutive transitions, dependent on the field strength. We also predict different dynamics of chain recovery in the presence and absence of a magnetic field in the direction of the chain. This prediction could be tested using electron microscopy imaging of cells crosslinked at different time points after the rupture of the chain by an external field or by live-cell imaging of cells with fluorescently labeled magnetosomes.

Overall, our simulations confirm the diversity of structure into which magnetic nanoparticles may assemble without attachment to a filament, which has been explored in previous studies. The presence of a filament to which the particles may bind stabilizes linear chain structures as required for the function as a cellular compass needle, for which the filament provides the needed mechanical stability. In addition, however, our simulations also underscore the dynamic nature of these structures, which may be even greater in cells, where the cytoskeletal filaments themselves are also dynamic, an aspect that may be considered in future extension of the current work. We should note that in our model we have used the external magnetic field as an external stimulus to apply mechanical forces to the magnetosome chain and explore its structure. However, in nature, the earth magnetic field that the bacteria experience (50*μ*T) is much smaller than the field strengths used here. Even if bacteria were immobilized, it would not cause disruption of the chain. Thus rather than studying the response of these cells to external fields they encounter in their natural environment, these experiments use the magnetic field as a tool to explore the mechanical structure of the cell’s interior. In that respect, they may be considered as a model system to understand the physical basis of magnetogenetics [30].

## Supporting information

S1 File Dataset of raw data for figures of the paper. (TXT)

S1 AppendixCritical angle for rupture.In section 1.2, we obtained the critical angle of the external field where *E*_*binding*_ > *E*_*elasticity*_ and as a result, the linker of the particle to the filament breaks. In this appendix, we look at this critical angle in more detail. To that end, we compare the energy of one unbinding particle in the bound and unbound configuration as indicated in Fig 4 (the other particle is considered as a provider of the dipole-dipole interactions). In the bound configuration, the energy is given by
$$\begin{array}{c} E_{bound}=E_{B}+E_{dd}+E_{elasticity}+E_{binding}. \end{array}$$(5)
After unbinding the energy includes only the external magnetic field-dipole and dipole-dipole interactions,
$$\begin{array}{c} E_{unbound}=E_{dd}+E_{B}. \end{array}$$(6)
We take the two magnetic moments to be parallel and aligned with the field and calculate both energies. Assuming that the linker breaks, when the bound energy exceeds the unbound one, we obtain the condition that the particle unbinds, when *E*_*binding*_ > *E*_*elasticity*_.
$$\begin{array}{c} \begin{array}{cc} & E_{elasticity}=+\frac{1}{2}k_{l}{\left(l-l_{0}\right)}^{2}\\ & l^{2}={\left(R+\frac{d}{2}\right)}^{2}+{\left(l_{0}+R+\frac{d}{2}\right)}^{2}+2l_{0}\left(R+\frac{d}{2}\right)\cos\left(\theta \right) \end{array} \end{array}$$
Lengths *l* and *l*_0_ are presented in S1 Fig. *E*_*binding*_ > *E*_*elasticity*_ leads to,
$$\begin{array}{l} {\left({\left({\left(R+\frac{d}{2}\right)}^{2}+{\left(l_{0}+R+\frac{d}{2}\right)}^{2}+2l_{0}\left(R+\frac{d}{2}\right)\text{cos}\left(\theta \right)\right)}^{\frac{1}{2}}-l_{0}\right)}^{2}\geq \left(\frac{2E_{b}inding}{k_{l}}\right)\\ \Rightarrow \;\text{cos}\left(\theta \right)\geq \frac{R+\frac{d}{2}}{2\left(R+\frac{d}{2}+l_{0}\right)}+\frac{R+\frac{d}{2}+l_{0}}{2\left(R+\frac{d}{2}\right)}-\frac{{\left(\sqrt{\frac{2E_{binding}}{k_{l}}}+l_{0}\right)}^{2}}{2\left(R+\frac{d}{2}\right)\times \left(R+\frac{d}{2}+l_{0}\right)} \end{array}$$
$$\begin{array}{ccc} & & \cos\theta \geq \frac{R+\frac{d}{2}}{2(R+\frac{d}{2}+l_{0})}+\frac{R+\frac{d}{2}+l_{0}}{2(R+\frac{d}{2})}-\frac{{\left(\sqrt{\frac{2E_{binding}}{k_{l}}}+l_{0}\right)}^{2}}{2\left(R+\frac{d}{2}\right)\times \left(R+\frac{d}{2}+l_{0}\right)} \end{array}$$(7)
Using the values from section 0.1, this leads to *θ* ≳ 27°.(ZIP)

S1 Fig The torque exerted by the external field pulls the linker of the particle to the filament with relaxed length *l*_0_ to the length *l*. (EPS)
